# Pregnancy Effect on Echocardiographic Parameters in Great Dane Bitches

**DOI:** 10.3390/ani10111992

**Published:** 2020-10-29

**Authors:** Monica Melandri, Ilaria Spalla, Luca Fanciullo, Salvatore Alonge

**Affiliations:** Ambulatorio Veterinario “Il Melograno”, Sesto Calende, 21018 Varese, Italy; drmonicamelandri@gmail.com (M.M.); illispa@hotmail.com (I.S.); lucaecovet@libero.it (L.F.)

**Keywords:** bitch, cardiac function, echocardiography, Great Dane, pregnancy

## Abstract

**Simple Summary:**

In women, as well as in bitches, pregnancy is associated with the adaptation of the left ventricular function. Pregnancy mainly represents a status of increased volume load of the maternal heart driven by the need to supply an adequate blood flow through an augmented uterine perfusion to the developing fetuses. Consequently, cardiac morpho-functional changes are expected. Cardiac morphology and function in pregnant bitches that would develop complications may differ from those with a normal outcome. Thus, the assessment of the maternal cardiovascular function may be a useful screening tool to detect pregnancy complications in dogs. Few studies investigated the cardiac maternal adaptation in different gestational ages in dogs. Breed is a known source of variation in echocardiographic values; hence, the cardiac performance during pregnancy may also be prone to a breed-related variation. For these purposes, due to possible breed-related variations in cardiac parameters and function, and to the specific predisposition for myocardial diseases, echocardiographic changes along pregnancy in healthy Great Dane bitches were evaluated. A significant effect of the age of gestation on the increase of functional and diastolic parameters and on the decrease of systolic parameters was reported.

**Abstract:**

Pregnancy is associated with adaptation of the left ventricular (LV) function. Due to differences between breeds in baseline echocardiographic values and specific predispositions for myocardial diseases, breed-specific echocardiographic parameters may be helpful to evaluate whether the systolic function varies during pregnancy. This study enrolled nine healthy Great Dane bitches with uncomplicated pregnancy. Echocardiographic M-mode and B-mode data were collected before ovulation and within 7 days of the predicted parturition term. Evaluated parameters were: LV dimension in diastole (LVd) and systole (LVs), end-diastolic (EDVI) and end-systolic (ESVI) volumes indexed to body surface area (BSA), end-diastolic (EDV) and end-systolic (ESV), end-point-septal-separation (EPSS), left atrium to aortic root ratio (LA/Ao), sphericity index (SI), ejection fraction (EF), fractional shortening (FS), stroke volume (SV), heart rate (HR), and cardiac output (CO). The ANOVA showed a statistical effect of the age of gestation (*p* < 0.01) on the increase of diastolic dimensions and functional parameters and on the decrease of systolic dimensions. The CO increase parallels the rise in SV and HR (*p* < 0.01). No statistical differences were observed for EPSS, LA/Ao, and SI. The changes in cardiac chambers and function are likely to reflect maternal adaptation to allow the fetal development in uncomplicated pregnancy. The present study provides specific echocardiographic values in uncomplicated pregnancy of Great Danes, showing that the systolic function is enhanced and that the increase in preload, observed during gestation, is the likely mechanism.

## 1. Introduction

Pregnancy is associated with an adaptation of the cardiac function. Several studies in pregnant women have demonstrated that hemodynamic adaptations to gestation reversibly affect maternal echocardiographic variables [1]. Pregnancy mainly represents a state of increased volume load of the maternal heart driven by the need to supply an adequate blood flow to the developing fetuses. The cardiac output and preload indicators, such as ventricular volume and left atrial size, increase progressively [2,3,4,5,6,7,8].

The cardiac adaptation to pregnancy was investigated in some species. In goats, for instance, gestation led to an increase in heart rate and cardiac output, while the chamber morphology and systolic function did not vary [9]. In Guinea pigs, either really pregnant or hormonally treated to mimic pregnancy, the augmented intravascular volume, the cardiac output and the stroke volume were reported, while the heart rate remained unchanged [10].

Several studies investigated cardiac maternal adaptation in different gestational ages in dogs, but, in comparison to women, only little information is available on morphological and functional echocardiographic parameters during pregnancy, sometimes with controversial conclusions [11,12,13,14,15,16,17,18]. The study by Abbott [12] highlighted increased heart rate (HR), cardiac output (CO), and systolic function indices of the left ventricle during the third trimester. Abbott performed an echocardiographic evaluation of bitches after parturition, concluding that the cardiac adaptation to pregnancy is reversible in dogs, too [12]. A decrease in the afterload and an increase in the systolic function assessed by fractional shortening (FS) and ejection fraction (EF) were reported in previous studies [15,16]. Such variations were reported in the third trimester and were considered to reflect pregnancy-related hemodynamic changes [15,16]. In the study by Blanco and colleagues [16], the FS, only, began to increase in the second trimester. Conversely, two recent studies reported the complete absence of echocardiographic variations in bitches from proestrus, through early and late pregnancy, to anestrus [17,18]. Previous studies enrolled mainly mixed or various breed dogs [10,12,14,17,18]. Breed is a known source of variation in echocardiographic values [19,20]; hence, the cardiac performance during pregnancy may also be prone to a breed-related variation.

Breed-specific echocardiographic reference values have been published in healthy Great Dane dogs [21,22,23]. This breed is at risk for dilated cardiomyopathy (DCM) and screening is advised, following the Consensus of the European Society of Veterinary Cardiology (ESVC). The currently accepted method for screening is echocardiography alongside with Holter monitoring [24,25]. The DCM may become evident on echocardiography by the presence of a dilated, hypokinetic left ventricle; in some dogs, arrhythmia or congestive heart failure may be present, too. Nevertheless, a final diagnosis can sometimes be challenging, mainly in subclinical statuses or when equivocal screening test results are achieved; thus, in some cases, sudden death can be the only evidence of the disease [20,25,26,27,28].

Finally, since the maternal cardiac adaptation during gestation plays a major role in uterine perfusion to support fetal development, the characterization and understanding of the maternal cardiac function during pregnancy is clinically important to recognize possible complications, from both the cardiac and obstetric point of view. On one hand, maternal heart diseases can be associated with non-obstetric mortality during pregnancy [29]. On the other hand, cardiac morphology and function in pregnant bitches that would exhibit complications may differ from those with a normal outcome. Particularly, Blanco and colleagues [13] compared echocardiographic parameters gathered during pregnancy from normal and abnormal canine pregnancies and reported that left ventricular dimension in diastole (LVd), HR, FS, Systolic Volumes (SV), and CO significantly differed between their two study groups, identifying a possible cardiac maladaptation to pregnancy in bitches with an abnormal gestational course. Thus, the assessment of the maternal cardiovascular function and morphology, with the description of pregnancy-related breed-specific physiological variations, may be a useful screening tool to detect pregnancy complications in dogs.

For these purposes, due to possible breed-related variations in the cardiac parameters and function, to the predisposition for myocardial diseases, and to the challenges in identifying subclinical statuses, it may be useful to evaluate echocardiographic changes along pregnancy in healthy Great Dane bitches having a normal pregnancy.

## 2. Materials and Methods

### 2.1. Ethics

The present study was performed in accordance with the ethical guidelines of the animal welfare committee. Procedures with animals were performed following good veterinary practice for animal welfare according to the Italian legislation on animal care (DL 116, 27/01/1992) and the European Guidelines on Animal Welfare (Directive 2010/63/EU).

Owner’s informed consent on the whole procedure was obtained.

### 2.2. Animals

The study enrolled nine healthy Great Dane bitches (2.5 to 4 years). Due to the breed predisposition to develop DCM, all of the bitches underwent an echocardiographic screening exam and Holter monitoring before being enrolled in the study. All of the bitches were proven to be clinically healthy; systolic blood pressure was within reference ranges and no murmurs were detected during auscultation; the echocardiographic exam displayed left ventricular size, function, and Doppler parameters within reference ranges for breed and weight [22,23,24]; Holter monitoring results were within normality ranges for each patient enrolled [24,25]. No clinical signs compatible with hypothyroidism and normal thyroxine levels (T4 reference range 23–50 nmol/L) were required to be included in the study. Total thyroxine was measured by an immunochromatographic quantitative test by laser-induced fluorescence analysis (Speed T4, Virbac, Milan, Italy).

For breeding management, the day of ovulation was considered when the serum progesterone concentration ranged between 4–10 ng/mL [30,31,32], as evaluated using an Enzyme Linked Fluorescent Assay (MiniVidas, BioMerieux, Marcy l’Etoile, France). Bitches were mated once, 2 days after the estimated ovulation day [32].

The day of parturition was estimated on the basis of several cumulative parameters. They were represented by blood progesterone concentrations (MiniVidas, BioMerieux) measured during heat [31], prediction of the parturition day, estimated by fetal biometry [33,34], and the confirmation of approaching parturition by blood progesterone concentrations <2 ng/mL [35]. Twenty days after mating, the inner diameter of the chorionic cavity (ICC) was measured, and in late pregnancy (around 40 days after mating) the biparietal diameter (BP) was assessed. At least three measurements of ICC or BP, according to the gestational period, were recorded and the mean values were calculated [34].

Together with cardiac health, a requisite for the inclusion in the present study was an uncomplicated pregnancy. The latter definition includes: successful delivery of full term puppies; absence of embryonic or perinatal death and stillbirth; normal-weighted puppies; absence of any maternal disorder of the reproductive sphere (i.e., pre-eclampsia, intra-uterine growth restriction, placental abruption) [13].

### 2.3. Procedure

Standard echocardiographic M-mode and B-mode data [36] were collected twice. The first echocardiographic evaluation was performed before ovulation, on day 0 of the estrus cycle (T0) [37]. The time to be identified with T0 was chosen following previous reports about echocardiography during pregnancy [12,13,14,16]. The second evaluation was performed 7 days before the predicted parturition term (T1), as the majority of previous reports on this topic concluded that the main echocardiographic variations during pregnancy occur in the third trimester of gestation [12,15,16]. Due to the same reason, the first and second trimester of gestation were not taken into account in the present study. For all of the bitches, the actual day of parturition overlapped with the estimated parturition term, thus T1 can be considered 7 days before delivery for all the subjects. On the same occasions, bitches went through a complete clinical visit; their general and cardiovascular objective examinations always reported normal parameters; heart murmurs were never detected.

For the ultrasonographic evaluation, dogs were gently restrained in lateral recumbency. An acoustic gel was applied to the transducer and coupled directly to the clipped thorax skin. No sedation was required. Grey-scale real-time ultrasound images were produced using a 1.5–5 MHz probe with a cardiac setting (My Lab 30 Gold, Esaote Spa, Genoa, Italy). The M-mode parameters included: left ventricular internal dimensions in systole (LVs) and diastole (LVd), obtained from a right parasternal short axis view at the papillary muscles, and end-point-septal-separation (EPSS), from a right parasternal short axis view at the mitral valve [38]. The B-mode measurements included: left atrial size to aortic root ratio (LA/Ao) using the Swedish method, end-diastolic volumes (EDV) and end-systolic volumes (ESV), end-diastolic (EDVI) and end-systolic (ESVI) volumes indexed to Body Surface Area (BSA). The Body Surface Area was calculated as follows: BSA = (10.1 × BW^2/3)/10^4, where BW stands for bodyweight. With regards to parameters assessing the systolic function, the fractional shortening (FS) was recorded from M-mode measurements, while the ejection fraction (EF) was calculated as previously described [39]. The cardiac output (CO) was calculated as the product of stroke volume (SV) and heart rate (HR). The sphericity index (SI) was calculated as the ratio between the length of the left ventricle to its width [38].

A simultaneous electrocardiogram recording was gathered while performing echocardiographic measurements [40].

Each parameter was measured three times, from consecutive cycles acquired by the operator; the mean value obtained from the three measurements was then used in the statistical analysis for each parameter referring to each enrolled patient [36,41,42].

### 2.4. Statistical Analysis

The normality distribution of all the echocardiographic parameters was verified by the Shapiro–Wilk test and data are presented as mean ± standard deviation. Changes of the ultrasonographic parameters along pregnancy were statistically evaluated by ANOVA repeated measures and the Tukey’s Honestly Significant Difference (HSD) test. Results were considered significant for *p* ≤ 0.05. The statistical analysis was performed with the online tool VassarStats: website for statistical computation (http://vassarstats.net, Vassar College, New York, NY, USA).

## 3. Results

All of the nine healthy bitches first enrolled in the study successfully delivered full term puppies with no gestational complications. Their clinical reports are summarized in Table 1. All of the puppies were alive. The median litter size was 8 live puppies (mean 7.11 ± 3.30; range 2–12 puppies). The birth weight was within the normal range reported in literature for this breed (minimum birth weight 400 g, maximum birth-weight 960 g; mean value 685 ± 171.7 g) [43]. Thus, all the bitches enrolled for the cardiac screening before and during gestation were kept in the study. The bodyweight (BW) of the nine bitches was 56.89 ± 3.86 kg at T0 and was 66.33 ± 5.34 kg at T1 (Table 1). The body surface area increased from 1.49 ± 0.07 m^2^ at T0 to 1.65 ± 0.09 m^2^ at T1 (Table 1).

As reported in Table 2, an increase in the diastolic dimensions (LVd, EDVI, and EDV) and a decrease in the systolic ones (LVs, ESVI and ESV) were observed at T1 (*p* < 0.01).

The functional parameters (FS, EF, CO, SV, and HR) significantly increased at T1 (*p* < 0.01) (Table 3). No statistical differences were observed for EPSS, LA/Ao and SI between T0 and T1 (Table 4).

At both time points, all of the echocardiographic parameters taken into account in the present study remained within the reference ranges reported for normality in the Great Dane breed (Table 2, Table 3 and Table 4) [21,23].

## 4. Discussion

The present study shows that some echocardiographic variables undergo statistically significant changes along uncomplicated pregnancies.

### 4.1. Changes in Morphological Parameters

The findings of the present study are consistent with previous reports about the physiological canine gestation, where LVd increase and LVs decrease was observed during the second half of gestation (Table 2) [15,16], but the values for LVd and LVs were still within the reference values for breed [21,23].

#### 4.1.1. Systolic Morphological Parameters

The results of this study demonstrate that systolic morphological parameters (LVs, ESVI, and ESV) significantly decrease in the third trimester of gestation (Table 2); the values were still within the reference values reported for the breed [21,23].

The evaluation of the systolic function is really complex, as it is affected from many factors (including preload, afterload, contractility, distensibility, coordinated contraction and heart rate) [39]. However, echocardiography can provide indirect parameters to aid in the estimation, including left ventricular systolic dimensions and volumes, which represent the ability of the left ventricle (LV) to pump an adequate amount of blood for every cardiac cycle. Based on our study results, no systolic function deteriorations were found in uncomplicated pregnancy, as demonstrated by the mild decrease in systolic diameters and volumes (Table 2), in contrast with what expected in DCM, where an increase in systolic volumes is one of the diagnostic criteria [24].

#### 4.1.2. Diastolic Morphological Parameters

The results of this study demonstrate that, during the physiologic adaptation to pregnancy, diastolic morphological parameters (LVd, EDVI, and EDV) significantly increase in the third trimester of gestation (Table 2); the values were still within the reference values reported for the breed, except for EDVI that slightly exceeded the normality ranges [21,23].

The increase in diastolic internal diameters and volumes (Table 2) reflects an increase in preload by the augmented circulating volume. The latter contributes to the rise in contractility secondary to the Frank-Starling mechanism (an increase in preload will increase intrinsic cardiac contractility in a healthy heart). Although anemia has been described during canine pregnancy [44], the mild decrease in hematocrit is unlikely to be responsible for fluid retention and increased preload; however, it is not possible to fully rule out that this aspect might play a minimal contributing effect [45].

### 4.2. Changes in Functional Parameters

The results of this study demonstrate that, during the physiologic adaptation to pregnancy, the functional parameters (FS, EF, CO, SV, and HR) significantly increase in the third trimester of gestation (Table 3), but still remain within the reference values reported for the breed [21,23], except for HR.

Previous studies showed that during pregnancy, a rise in cardiac output, due to augmented circulating blood volume and decreased systemic vascular resistance, is generally expected in women [46,47] and strongly suspected in bitches, too [15,16]. As CO is the result of stroke volume and heart rate [39], it is not surprising that both these parameters were increased, contributing to the rise in the cardiac output observed during the last trimester of pregnancy (Table 3). All of these parameters indicate an increased blood delivery to meet the augmented body demands and fetal blood supply.

Heart rate is the parameter that can most easily be monitored [48]. Previous studies showed that HR was higher in dogs with normal pregnancy, while a lack of hemodynamic adaptation was observed in abnormal gestation that did not show the expected HR increase [13,49]. Similar results were reported in women, where HR was higher in patients with normal uterine perfusion in comparison to those with abnormal pregnancies [50].

The increase in the ejection fraction (Table 3) also indicates an increase in the heart pumping capacity, likely due to the increase in preload and end-diastolic volumes and to the decrease in the systemic vascular resistance and end-systolic volumes (afterload). A significant EF increase was observed in normal human pregnancies, too, but not in complicated ones [51]: a lack in EF increase, which is a marker of the left ventricular systolic function, could in fact indicate an altered cardiovascular response to the volume overload linked to pregnancy.

Previous studies reported a slightly higher ejection fraction in normal than in complicated canine gestation from the thirtieth day onwards [12,13]. The EF increase is likely the result of the combined increased preload and decreased afterload, typical of pregnancy [39], as illustrated by the increased diastolic dimensions and the decreased systolic ones (Table 2).

### 4.3. No Changes in Echocardiographic Parameters: EPSS, LA/Ao, SI

The results of this study demonstrate that, during the physiologic adaptation to pregnancy, EPSS constantly remains within the normal range (<6 mm) (Table 4) [38,52], indicating that the hemodynamic changes in pregnancy do not alter this parameter, in contrast with DCM, where EPSS is expected to increase.

Several studies suggested that the assessment of LV volume and shape could allow to early detect dogs at risk for rapid progression into heart diseases [38,53,54,55,56]. The sphericity index aims to recognize the geometrical change of LV when it dilates due to cardiac diseases, such as DCM, and so that the heart acquires a rounder shape [38,57,58]. In the present study, no differences were observed in SI along pregnancy (Table 4): the increase of cardiac activity in gestation, even if associated with a rise in diastolic dimensions and a physiologic volume overload, is not associated with any spherical dilatation, which remains associated to pathologic conditions (i.e., DCM) [59].

The LA/Ao did not significantly change along pregnancy and, as already described in literature, it remained around 1.1 (Table 4) [60] and within the normal reference range published for healthy Great Danes [21,23], supporting what previously stated about EPSS and SI.

### 4.4. Clinical Relevance

Previous authors recently suggested that the cardiac function and morphology of pregnant bitches, prone to develop complications, are different from those with a normal uncomplicated pregnancy [14]. Therefore, it seems important to underline that pregnancy-related breed-specific physiological variations should be known, especially in purebred dogs predisposed to heart diseases. Regardless of the echocardiographic evaluation during pregnancy, the pre-requisite for breeding should include cardiac screening to enroll healthy bitches, only, using the available echocardiographic criteria and breed-specific reference ranges. The assessment of the maternal left ventricular structure and function, with the evidence of an adaptation differing from the expected one, may provide important prognostic information. Furthermore, the echocardiographic values obtained before the planned pregnancy could provide basal values to be compared with those recorded during gestation in order to promptly identify any possible maternal maladaptation. The results of the present study indicate that an increase in diastolic dimensions (Table 2) and functional parameters (Table 3) and a decrease in systolic dimensions (Table 2) should be expected in uncomplicated pregnancy in healthy Great Dane bitches, while EPSS, LA/Ao, and SI should remain unchanged (Table 4). In healthy Great Dane bitches, the volume overload and the hemodynamic alterations of pregnancy are not able to induce variations in EPSS, LA/Ao, and SI, which, with today’s knowledge, remain typical features of cardiac diseases, i.e., DCM. An abnormal cardiovascular adaptation would precede the appearance of late complications [14]. This aspect still needs to be described in Great Dane bitches affected by DCM, even at subclinical stages.

The results of the present study are in accordance with previous researches reporting echocardiographic variations of morphological and functional parameters during the third trimester of pregnancy [11,12,13,14,15,16]. Those echocardiographic measurements that statistically changed in the third trimester from T0 baseline always remained within reference ranges of normality for the breed [21,23], such as in the study by Abbott [12]. This aspect seems to be of great clinical relevance: in healthy bitches, pregnancy is able to induce some significant cardiovascular alterations, but echocardiographic values do not exceed reference ranges as a normal healthy heart is able to compensate the physiologic volume overload through its function and morphology. Blanco and colleagues reported that, conversely, in abnormal canine pregnancy the values undergoing variations in the third trimester of a normal pregnancy do neither change nor even oscillate towards an opposite direction [13]. Unfortunately, the latter study does not compare the results obtained from abnormal pregnancies with normal reference values as the bitches enrolled were of different breeds and size [13]. The present study has its strength in the consistent evaluation of dogs of the same breed, allowing the comparison of gathered results to existing normal reference values. The results of the present study suggest that there should be no need to exclude giant breeds from echocardiographic examinations even in the third trimester of gestation because recorded parameters remain still in reference values for the breed [21,23] despite the physiological adaptation to pregnancy. However, before suggesting to routinely perform echocardiographic evaluations during pregnancy to state the maternal healthy cardiac condition, it would be interesting to compare the present results gathered from pregnant Great Dane bitches completely free from cardiac diseases (even at subclinical statuses), with data similarly collected from Great Dane bitches affected by cardiovascular alterations to verify whether the variation trends reported in this study would be absent in ill Great Danes, as suggested in various breed dogs by Blanco and colleagues [13], or whether cardiac parameters would exceed reference ranges reported for normality. Similar evaluations could be applied to bitches of different breeds, predisposed to the same or to different cardiac disorders.

The present results are in contrast with studies concluding that no cardiac alterations can be detected from estrus to post-partum anestrus throughout the whole pregnancy [17,18]. Some possible explanations were already suggested by Ward and colleagues [18]. The patients enrolled were quite different in terms of breeds and size: the present study enrolled giant-breed dogs (Great Danes), only, while the previous one included mainly medium-size subjects (German Shepherd, Golden Retriever, Vizsla, Weimaraner) and even small ones (Yorkshire Terrier) [18]. Bitches from different breeds could have specific cardiac adaptations to pregnancy. Moreover, the patients enrolled in the previous study were reported to be unfamiliar with the hospital environment, this factor possibly increasing the sympathetic tone and affecting some echocardiographic evaluations [18]. Conversely, the bitches evaluated in the present study were very familiar with the veterinary environment and with the practitioners performing procedures. The same explanation could be valid for the total absence of heart murmurs at any time point of the present study. Finally, Ward and colleagues [18] hypothesize that the intense activity of the sympathetic nervous system described for their patients could have biased the advanced imaging modalities they used, such as strain imaging algorithms and three-dimensional volumetric calculations [18]. However, in the present study, a statistically significant difference was observed between parameters recorded before pregnancy and during the third trimester of gestation, but, similarly to reports from a previous study [18], the echocardiographic references values for the breed [21,23] were noticed even in the third trimester of gestation.

Further studies would be advisable to verify exactly when, after parturition, the cardiac morphology and function return to pre-gestational values, in order to confirm that in dogs, as reported in humans, pregnancy promotes a reversible maternal cardiac adaptation with no long-term effects on the cardiac function [1,61,62]. Echocardiographic evaluations in anestrus following parturition were performed in two previous studies, only. In the research by Abbott [12], the post-partum examination was performed 8 weeks after parturition, when the variables undergoing changes during the last trimester of gestation had already returned to basal values. This investigation was not able to precisely state when, after parturition, cardiac morphology and function reach pre-gestational levels again: further studies should perform weekly cardiac evaluations after delivery. Conversely, Ward and colleagues [18] reported the absence of any statistically significant difference in the echocardiographic parameters between proestrus and anestrus, considered to occur between 75 and 120 days after parturition. However, the results of the latter study cannot be fully compared to the present ones, as the authors observed no significant echocardiographic changes at any phase of the cycle [18].

Finally, the present study does not report data from the first and second trimester of gestation. Those periods were not taken into account as time points to perform echocardiographic evaluations as all the previous studies highlighting cardiac changes during pregnancy reported significant changes towards the third trimester, only [12,13,14,16].

## 5. Conclusions

The need for adequate blood supply to support the fetal development is associated with maternal cardiac changes, including an increase in the preload and in the systolic function, alongside with an increase in the left ventricular dimensions in diastole and a reduction in systole. As a consequence, cardiac output, stroke volume, and heart rate increase. The results of the present study suggest that, even though pregnancy represents a cause of physiological maternal volume overload [44], the cardiac adaptation in bitches with a healthy cardiovascular system is not associated with a decreased systolic function.

## Figures and Tables

**Table 1:** Clinical reports of the enrolled Great Dane bitches at T0 and T1, expressed as mean ± SD.

Observed Parameter	Time 0	Time 1
Bodyweight (kg)	56.89 ± 3.86	66.33 ± 5.34
BSA (m^2^)	1.49 ± 0.07	1.65 ± 0.09
Heart rate (BPM)	100.41 ± 10.99	131.00 ± 16.47
Heart murmurs (%)	0	0

SD = standard deviation. BSA = body surface area. BPM = beats per minute.

**Table 2:** Morphological parameters changing along pregnancy, expressed as mean ± SD at T0 and T1. The last column reports echocardiographic reference ranges of normality in Great Danes for each parameter considered (Koch et al. [21]; Stephenson et al. [23]).

Variable	Time 0	Time 1	Normality Range Mean Value (Min–Max) [21] Mean Value (5–95 Percentile) [23]
LVd (mm)	51.34 ± 3.58 ^a^	54.36 ± 2.67 ^b^	54.00 (44.00–59.00) [21] 52.00 (42.70–58.70) [23]
LVs (mm)	36.54 ± 2.67 ^a^	35.84 ± 2.65 ^b^	39.50 (34.00–45.00) [21] 38.00 (28.80–42.50) [23]
EDVI (mL/m^2^)	85.11 ± 14.87 ^a^	87.42 ± 12.83 ^b^	82.00 (54.00–95.00) [21]
ESVI (mL/m^2^)	36.44 ± 6.44 ^a^	34.45 ± 6.06 ^b^	42.00 (26.00–55.00) [21] 36.50 (21.90–47.00) [23]
EDV (mL)	107.31 ± 19.05 ^a^	115.28 ± 18.34 ^b^	
ESV (mL)	50.81 ± 8.20 ^a^	49.80 ± 9.56 ^b^	

Different superscripts denote statistically significant differences between the two time-point columns (ANOVA *p* < 0.01). SD = standard deviation. T0 = time 0. T1 = time 1. LVd = left ventricular internal dimension in diastole. LVs = left ventricular internal dimension in systole. EDVI = end-diastolic volume index. ESVI = end-systolic volume index. EDV = end-diastolic volume. ESV = end-systolic volume. ANOVA = analysis of the variance.

**Table 3:** Functional parameters and their adaptation along pregnancy, expressed as mean ± SD at T0 and T1. The last column reports echocardiographic reference ranges of normality in Great Danes for each parameter considered (Koch et al. [21]; Stephenson et al. [23]).

Variable	Time 0	Time 1	Normality Range Mean Value (Min–Max) [21] Mean Value (5–95 Percentile) [23]
EF (%)	56.56 ± 6.00 ^a^	60.04 ± 5.31 ^b^	48.00 (33.00–65.00) [21] 54.50 (42.10–63.90) [23]
FS (%)	29.96 ± 4.13 ^a^	32.59 ± 3.66 ^b^	25.00 (18.00–36.00) [21] 27.00 (20.00–37.00) [23]
SV (mL)	62.24 ± 9.57 ^a^	68.61 ± 12.93 ^b^	
HR (bpm)	100.41 ± 10.99 ^a^	131.00 ± 16.47 ^b^	110.00 (100.0–130.00) [21]
CO (L/min)	6.31 ± 1.79 ^a^	8.45 ± 1.68 ^b^	

Different superscripts denote statistically significant differences between the two time-point columns (ANOVA *p* < 0.01). SD = standard deviation. T0 = time 0. T1 = time 1. EF = ejection fraction. FS = fractional shortening. SV = stroke volume. HR = heart rate. BPM = beats per minute. CO = cardiac output. ANOVA = analysis of the variance.

**Table 4:** Parameters that do not statistically change along pregnancy, expressed as mean ± SD at T0 and T1. The last column reports echocardiographic reference ranges of normality in Great Danes for each parameter considered (Koch et al. [21]; Stephenson et al. [23]).

Variable	Time 0	Time 1	Normality Range Mean Value (Min–Max) [21] Mean Value (5–95 Percentile) [23]
EPSS (mm)	5.08 ± 1.55 ^a^	5.30 ± 1.66 ^a^	8.00 (5.00–12.00) [21] 5.60 (3.00–8.60) [23]
LA/Ao	1.12 ± 0.09 ^a^	1.11 ± 0.55 ^a^	1.10 (0.90–1.50) [21] 1.19 (0.91–1.41) [23]
SI	1.70 ± 0.05 ^a^	1.75 ± 0.08 ^a^	1.70 (1.51–2.00) [23]

Equal superscripts denote the absence of statistically significant differences between the two time-point columns (ANOVA *p* > 0.05). SD = standard deviation. T0 = time 0. T1 = time 1. EPSS = E-point to septal separation. LA/Ao = left atrium to aortic root ratio. SI = sphericity index. ANOVA = analysis of the variance.
